# Peptide-Enriched Silk Fibroin Sponge and Trabecular Titanium Composites to Enhance Bone Ingrowth of Prosthetic Implants in an Ovine Model of Bone Gaps

**DOI:** 10.3389/fbioe.2020.563203

**Published:** 2020-10-19

**Authors:** Arianna B. Lovati, Silvia Lopa, Marta Bottagisio, Giuseppe Talò, Elena Canciani, Claudia Dellavia, Antonio Alessandrino, Marco Biagiotti, Giuliano Freddi, Francesco Segatti, Matteo Moretti

**Affiliations:** ^1^IRCCS Istituto Ortopedico Galeazzi, Cell and Tissue Engineering Laboratory, Milan, Italy; ^2^IRCCS Istituto Ortopedico Galeazzi, Laboratory of Clinical Chemistry and Microbiology, Milan, Italy; ^3^Ground Sections Laboratory, Department of Biomedical, Surgical and Dental Sciences, University of Milan, Milan, Italy; ^4^Silk Biomaterials srl, Lomazzo, Italy; ^5^Limacorporate S.p.A., Villanova di San Daniele del Friuli, Italy; ^6^Regenerative Medicine Technologies Lab, Ente Ospedaliero Cantonale, Lugano, Switzerland

**Keywords:** prosthetic implant, titanium, osseointegration, silk fibroin, bone

## Abstract

Osteoarthritis frequently requires arthroplasty. Cementless implants are widely used in clinics to replace damaged cartilage or missing bone tissue. In cementless arthroplasty, the risk of aseptic loosening strictly depends on implant stability and bone–implant interface, which are fundamental to guarantee the long-term success of the implant. Ameliorating the features of prosthetic materials, including their porosity and/or geometry, and identifying osteoconductive and/or osteoinductive coatings of implant surfaces are the main strategies to enhance the bone-implant contact surface area. Herein, the development of a novel composite consisting in the association of macro-porous trabecular titanium with silk fibroin (SF) sponges enriched with anionic fibroin-derived polypeptides is described. This composite is applied to improve early bone ingrowth into the implant mesh in a sheep model of bone defects. The composite enables to nucleate carbonated hydroxyapatite and accelerates the osteoblastic differentiation of resident cells, inducing an outward bone growth, a feature that can be particularly relevant when applying these implants in the case of poor osseointegration. Moreover, the osteoconductive properties of peptide-enriched SF sponges support an inward bone deposition from the native bone towards the implants. This technology can be exploited to improve the biological functionality of various prosthetic materials in terms of early bone fixation and prevention of aseptic loosening in prosthetic surgery.

## Introduction

The increased incidence of osteoarthritis in elderly and sport amateurs can eventually require arthroplasty. Only in the United States, over 700,000 knee and 300,000 hip replacements are performed every year (Raphel et al., 2016). The long-term stability of the prosthesis is mandatory to avoid the failure of the primary implant due to aseptic loosening (Pedersen et al., 2005). Indeed, aseptic loosening after primary joint replacement is the major cause of shortened active life and reduced prosthetic stability, counting a revision rate in 18% of the cases (Kurtz et al., 2005, 2007; Bozic et al., 2010; Mcgrory et al., 2016; Delanois et al., 2017). Inflammatory wear particles, peri-acetabular osteolysis, and stress shielding have been reported at long-term follow-up with some prostheses (Jasty et al., 1994; Von Knoch et al., 1997; Urbanski et al., 2012; Raphel et al., 2016). Additionally, bone debridement in the case of revision surgery or oncologic resection generates an interfacial gap between bone and implant devices, which can seriously affect bone-implant osseointegration (Bozic et al., 2010; Capanna et al., 2015). Bone cements have been widely used in orthopedic surgery to grant implant fixation in the case of interfacial gaps. However, bone damages have been reported due to the thermal necrosis determined by cement polymerization (Stanczyk and Van Rietbergen, 2004). Hence, in the last years, the use of cementless joint replacement is increased, albeit cementless prostheses require a specific structure or bioactive coatings to guarantee a long-term implant fixation (Zhang et al., 2014). Several approaches have been proposed to optimize implant mechanical fixation by ameliorating the features of prosthetic materials (titanium or tantalum), such as porosity and/or geometry, to enhance the contact surface area to the bone (Simmons et al., 2001; Banerjee et al., 2013; Cheng et al., 2014). Many authors have demonstrated that a strong primary fixation with highly micro-porous metals can diminish the occurrence of bone loss and osteolysis (Mulier et al., 2006; Malizos et al., 2008). Alternatively, macro-porous metals have been used to generate acetabular cups and to fill cavitary bone defects, such as trabecular titanium (TT) that is characterized by multiple layers of hexagonal pores to resemble the structure of cancellous bone (Marin et al., 2010). Despite the great advantages provided by these novel structural modifications, severe gaps between implant-bone interfaces still represent an important clinical burden due to difficult bony bridging (Gruen et al., 2005; Malizos et al., 2008; Nakashima et al., 2013).

To overcome this limitation, coatings of implant surfaces have been studied for their osteoconductive and/or osteoinductive properties, such as hydroxyapatite and calcium phosphate (Kim et al., 2004; Chen et al., 2011; Zhang et al., 2014). Although some studies have shown that biomimetic calcium phosphate coatings can be precipitated from aqueous solutions to produce uniform layers on porous metals with favorable osteoconduction (Barrere et al., 2003; Lopez-Heredia et al., 2008), other studies have described the limitations related to inorganic coatings due to their interference with the interconnectivity of titanium pores that impedes the infiltration of resident cells within the implant (Chen et al., 2011). The enrichment of implants with specific growth factors and supplements has been also investigated to ameliorate cell and matrix ingrowth (Lamberg et al., 2006; Lu et al., 2012; Lopa et al., 2013; Lovati et al., 2015). However, local stimuli of growth factors to induce bone ingrowth are limited in a time-dependent manner due to their short biological half-life, usually limited to a few hours (Lee et al., 2011; Chettri and Nandi, 2012). Other coatings of micro-porous titanium implants have been investigated, such as RGD (arginine-glycine-aspartate) peptide solutions, used to bio-functionalize the metallic surface and promote implant osseointegration (Elmengaard et al., 2005). RGD peptides have also been combined with a silk-derived protein, the sericin, as a potential promoter of bone deposition within titanium implants (Zhang et al., 2008; Nayak et al., 2013). Yet, sericin is known to activate macrophages and induce inflammation, which represents a major limitation to its clinical use (Altman et al., 2003). Another silk protein, fibroin, has been proposed as a less inflammatory candidate for the restoration of bone defects thanks to its established biocompatibility and slow degradability (Zhao et al., 2009). The unique chemical, physical and mechanical features of silk fibroin (SF) are useful for inducing cells toward the osteogenic differentiation, as demonstrated by several promising *in vitro* and *in vivo* results about osteogenesis on silk materials (Midha et al., 2016). Regarding the role of silk matrices in inducing osteogenic-related signaling able to guide stem cells toward the osteogenic lineage, fibroin has been reported to induce the upregulation of alkaline phosphatase and other osteogenic markers, as well as the osteogenic differentiation of bone marrow cells (Jung et al., 2013). Enhanced osteoconductive properties can be achieved when SF scaffolds are loaded with bioactive agents (such as growth factors like bone morphogenetic protein 2, BMP-2), thus promoting accelerated bone deposition and healing (Karageorgiou et al., 2006; Jang et al., 2010). Nevertheless, the use of growth factors, in particular BMP-2, can be associated to severe adverse effects as inflammation and osteoclasts-mediated bone resorption (James et al., 2016). Recent *in vitro* studies have shown that anionic SF-derived polypeptides (Cs fraction, obtained by the chymotryptic cleavage of the SF chain) are able to nucleate carbonated hydroxyapatite when in contact with body fluids and accelerate the osteoblastic differentiation of murine bone marrow-derived stem cells in 3D osteoid-like dense collagen niches (Marelli et al., 2012, 2014). The osteogenic power of the Cs peptide fraction has been attributed to its electronegative potential deriving from the presence of specific amino acid sequences able to mimic the functionality of non-collagenous anionic proteins in the physiological process of biomineralization. These features and the demonstrated ability of Cs peptides to induce apatite deposition *in vitro* and accelerate osteogenic outcomes suggests that applying the Cs peptides in bone repair therapies may represent a promising approach (Griffanti et al., 2018).

The present study was conducted to assess whether the association of macro-porous TT with a SF sponge enriched with anionic fibroin-derived polypeptides (SF/Cs) – where SF acts both as a filler of the trabeculae of the titanium implants and as a carrier of Cs peptides – could improve early bone ingrowth into the implant mesh, thus taking advantage of the previously demonstrated osteogenic potential of the Cs peptides (Marelli et al., 2014). To this aim, implants were tested in a sheep model of cancellous bone defects with 1 and 3 mm bone-implant gaps around the implant. For this purpose, standard TT without fibroin was used as a reference and the new bone formation within the implant was evaluated after 2 months.

## Materials and Methods

### Study Design

Customized TT implants (Ti6Al4V) were designed and manufactured by Limacorporate S.p.A. (Villanova di San Daniele del Friuli, Italy). Prior to *in vivo* implantation, the TT mesh was filled with a SF sponge enriched with anionic fibroin-derived peptides (Cs) by means of a process developed by Silk Biomaterials Srl (Lomazzo, Italy). Six sheep were randomly implanted with the TT++SF/Cs composite devices in bone defects in femur and tibia to avoid a location bias, and then sacrificed after 2 months. Empty TT implants were used as controls.

### Preparation and Characterization of the TT+SF/Cs Composite Devices

A schematic description of materials and process is reported as Supplementary data (Supplementary Figure 1).

#### Trabecular Titanium Implant (TT)

**FIGURE 1:**
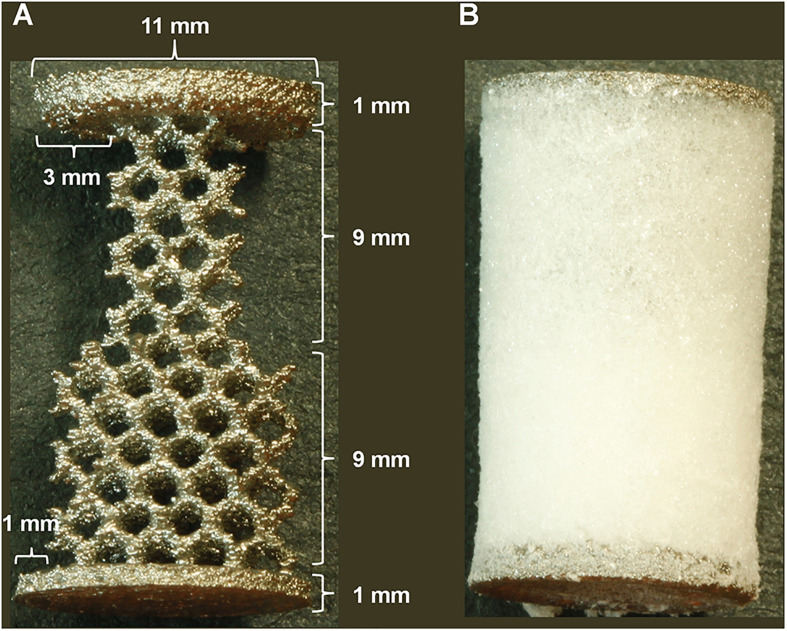
Implant design. **(A)** trabecular titanium (TT) implant dimensions with 3 and 1 mm concentric gap models. **(B)** TT implant incorporated into the peptide-enriched silk fibroin sponge [TT+ silk fibroin (SF)/Cs].

Customized TT implants were provided as cylinders (h 20 mm) with a trabecular structure and two compact heads at the edges (Ø 11 mm, h 1 mm; Figure 1A). The central part of the implant was divided in two 9 mm-high cylinders with different diameters (Ø 8 and Ø 10 mm) to generate 1 mm and 3 mm bone-implant gaps around the cylindrical implant.

#### Fibroin-Derived Anionic Peptides (Cs)

Cs fibroin-derived anionic polypeptides were prepared as previously described (Marelli et al., 2012). Briefly, an aqueous SF solution was treated with α-chymotrypsin (Sigma-Aldrich, Milan, Italy; enzyme-to-substrate ratio of 1:100) for 24 h at 37°C. The gelatinous precipitate was separated from the supernatant by centrifugation at 5,000 rpm for 20 min (Eppendorf, Hamburg, Germany). The anionic Cs peptide fraction was recovered in the powder form from the supernatant by freeze-drying.

#### Fibroin-Derived Cs Peptides-Enriched Silk Fibroin Sponges (SF/Cs)

Raw SF fibers were degummed in deionized water in autoclave at 120°C for 20 min, and extensively rinsed with warm and cold water to remove any sericin residues. Pure SF fibers thus obtained were dissolved in 9.3 M LiBr solution for 3 h at 60°C under mild agitation (SF concentration 10% w/v). The SF solution was then diluted with an equal volume of distilled water, filtered, and dialyzed for 3 days, with several water changes, until the complete removal of the salt. The final SF aqueous solution (precursor of the SF sponge) had a concentration of 2.5% w/v, as determined by evaporating a known volume of the solution and weighing the solid residue dried at 105°C for 2 h with an analytical balance. To obtain the Cs-enriched SF solutions, different amounts of Cs peptides, from 2.5% w/w up to 25% w/w with respect to the amount of SF in solution, were added. Neat SF sponges or SF/Cs sponges were obtained by pouring the respective solution in a mold and freezing at −20°C for at least 12 h, followed by freeze-drying.

#### Preparation of the TT+SF/Cs Composite Device (Italian Patent Application No. IT102020000004081)

The TT implant was placed into a transparent cylindrical mold (20 mm height; 12 mm diameter). The Cs-enriched SF solution was gently poured into the mold until the device was completely covered. After checking for the absence of air bubbles, the mold was put in a refrigerator at −20°C for at least 12 h to freeze the SF/Cs solution around and inside the TT structure. Afterward, the SF/Cs sponge was generated inside the TT structure by freeze-drying. The sponges incorporated into the TT device are shown in Figure 1B. After generating the TT+SF/Cs composites, they were sterilized by means of β-irradiation with intensities of 15 kGy using a 10 MeV source (Steris S.p.A., Seriate, Italy).

#### Chemical, Physical, and Morphological Characterization of the Materials

The amino acidic composition was determined by ion exchange chromatography, after acid hydrolysis with 6 N HCl at 105°C under vacuum, using an external standard for calibration.

Fourier transform infrared (FTIR) spectra were measured by the attenuated total reflection (ATR) mode with an ALPHA FTIR spectrometer (Bruker) equipped with a Platinum ATR diamond crystal, using a resolution of 4 cm^–1^, 24 scan acquisitions, and a spectral range of 4,000–400 cm^–1^.

Differential scanning calorimetry (DSC) thermograms were measured with a DSC 3500 Sirius (Netzsch) calorimeter, from room temperature to 400°C, at a heating rate of 10°C/min. Samples, 3–5 mg each, were put in an open aluminum pan and swept with N_2_ during the analysis.

Scanning electron microscopy (SEM) analysis was performed with EVO MA10 microscope (Zeiss), at 15kV, after sputter-coating with Au/Pd (Desk IV, Denton Vacuum, LLC).

### *In vitro* Studies of Cs Peptide-Enriched SF Sponges

#### Cytotoxicity Tests

3-(4,5 Dimethylthiazol-2-yi)-2,5 diphenyltetrazolinum bromide (MTT) assay was performed to assess the potential cytotoxicity of different concentrations of SF/Cs sponges. Briefly, National Institute of Health (NIH)-3T3 murine fibroblasts were seeded in the lower chamber of six-well plates at 3,000 cells/cm^2^ in Dulbecco’s modified Eagle’s medium high glucose (DMEM, Gibco, Life Technologies, Monza, Italy) added with 10% fetal bovine serum (FBS, Hyclone, Life Technologies, Monza, Italy), 100 U/ml penicillin-streptomycin, 2 mM L-glutamine, 1% sodium pyruvate, and 1% N-2-hydroxyethylpiperazine-N’-2-ethanesulfonic acid (HEPES) (all from Gibco, Life Technologies, Monza, Italy). Then, the SF sponges (Ø 5 mm × h 3 mm) enriched or not with different concentrations of peptides (0, 2.5, 5, 7.5, and 10% w/w) were placed into the upper chamber of the Transwell insert (pore Ø 3 μm, Greiner Bio-one) to perform an indirect culture. After 48 h, the SF sponges were removed and the cell viability was analyzed by MTT assay (Sigma-Aldrich, Milan, Italy). Briefly, 500 μl of MTT in DMEM without phenol red (0.5 mg MTT/ml) was added to the cell monolayer and incubated at 37°C for 4 h. After medium removal, formazan crystals were dissolved in 500 μl of HCl and isopropanol solution (1:10) for 10 min at RT under agitation. The absorbance was read at 570 nm with a multiplate reader (Perkin Elmer Victor X3). Data were reported as absorbance values and compared to cell treated with 0.1% Triton X-100 as a positive control (PC) and to cells cultured in fresh medium as a negative control (NC).

#### Live&Dead Assay

To evaluate the cell viability of NIH-3T3 fibroblasts, cells were directly seeded onto dry SF sponges enriched or not with 7.5 and 10% peptides and cultured for 48 h. Then, the Live&Dead Viability/Cytotoxicity test (Life Technologies, Monza, Italy) was performed by labeling the samples with 2.5 μl calcein AM and 10 μl ethidium homodimer-1 dissolved in 5 ml of PBS. After 30 min of incubation at RT, microphotographs were captured with an Olympus IX71 microscope and Olympus XC10 camera (Japan).

### *In vivo* Study of TT+SF/Cs Composite Devices

#### Ethics Statement

The whole study was approved by the Lazzaro Spallanzani Institute Animal Care and Use Committee (IACUC; Permit N. 454/2015-PR). The animals were housed at the Institute’s Animal Care Facilities that meet international standards. The Lazzaro Spallanzani Institute adheres to the principles set out in the following laws, regulations, and policies governing the care and use of laboratory animals: Italian Governing Law (D.lgs 26/2014) and EU directives and guidelines [European Economic Community (EEC) Council Directive 2010/63/UE]. The animals were regularly checked by a certified veterinarian responsible for health monitoring, animal welfare supervision, experimental protocols, and procedure revision. All surgeries were performed under general anesthesia, and all efforts were made to minimize suffering.

#### Surgical Procedure

Six skeletally mature female adult sheep (Bergamasca, 72 ± 10 kg body weight, mean age 3 ± 2 years old) were used. Surgery was performed as similarly described elsewhere (Lovati et al., 2016). Briefly, under general anesthesia, a cancellous bone defect (Ø 11 mm, depth 20 mm) was drilled into the proximal epiphysis of each tibia and into the distal epiphysis of each femur through a medial approach by means of a trephine under constant irrigation. The drilled holes were then implanted with differently treated TT customized implants, consisting of two overlapped 9 mm-high cylinders with two different diameters (Ø 8 and Ø 10 mm), as shown in Figure 1A. Thus, TT or TT+SF/Cs (enriched with 10% w/w Cs peptides) implants were inserted within the defects by press fit. The TT or TT+SF/Cs implants were allocated to each hole based on a 2 × 2 Latin square randomization. So, each animal received TT in the first site (i.e., right femur) followed by TT+SF/Cs in the second site (i.e., right tibia), and the reverse ordering in the other limb (left limb). The remaining three animals received TT+SF/Cs in the first site (i.e., right femur) followed by TT in the second site (i.e., right tibia), and the reverse ordering in the other limb (left limb). To verify the correct position of implants, a media-lateral fluoroscopic imaging (Cardiodigit 945, SIAS S.p.A.) was performed. Finally, wound layers were closed routinely. Antibiotics (enrofloxacin 5 mg/kg SC, Baytril 2.5%, Bayer; ampicillin 1 g IM, Amplital-Vet 20%, Ceva) and analgesics (meloxicam 0.4 mg/kg/die IV, Metacam, Boehringer Ingelheim) were administered postoperatively and for 7 days after surgery. After 2 months, animals were euthanized under deep anesthesia with an overdose of pentothal sodium (Thiopental, 30 mg/kg IV, Farmaceutici Gellini S.r.l.) and the hind limbs were explanted to carry out histological and SEM analysis.

### Histological and Histomorphometric Analyses

The explanted limbs were cut with a rotating saw in order to obtain 24 bone blocks, each containing one implant. Samples were processed for ground sections, as reported elsewhere (Donath and Breuner, 1982; Canullo et al., 2016; Ragone et al., 2020). Briefly, the blocks were immediately fixed in 10% formalin for 10 days, then washed in phosphate buffer saline (PBS, pH 7.4), dehydrated in increasing alcoholic scale, infiltrated in a solution of ethanol and acrylic resin and finally embedded in a light curing resin (Kulzer Technovit 7200 VLC, Exakt, Norderstedt, Germany) using a polymerization machine under vacuum (Exakt 520, Norderstedt, Germany). All the embedded specimens were then cut using a diamond blade (Micromet, Remet, Bologna) and glued on plastic slides with a specific gluing machine (Exakt 402, Norderstedt, Germany). Successively, the sections were grounded to a thickness of approximately 80–100 μm using a grinding system (Remet LS2, Bologna), stained with Toluidine Blue (Fluka, Taufkirchen, Germany) and counterstained with Pyronin Yellow (Sigma Aldrich, Italy). Toluidine Blue and Pyronin Yellow is a largely utilized bichrome staining indicated for methacrylate sections that allows to highlight bone matrix at different stages of mineralization (Canullo et al., 2016; Varoni et al., 2018; Ragone et al., 2020).

For each specimen, six equidistant sections perpendicular to the major axis of the scaffold were obtained: three in the portion with the 1 mm gap and three in portion with the 3 mm gap of the implant. Thus, a total of 144 slides were obtained in order to perform morphological and histomorphometric analyses.

All the slides were observed using a light microscope (Nikon Eclipse 80i, Japan) equipped with a digital camera (DXM1200, Nikon, Tokyo, Japan). In each specimen, an overview of the two most significant sections was acquired at total magnification of 20×. Also, a histomorphometric analysis was performed on each section using 10 photos taken from the central area of the scaffold at total magnification of 100× (Ragone et al., 2020). Finally, the inflammatory infiltrate, the amount of fibrosis, fatty infiltrate and necrotic areas were analyzed on images obtained at total magnification of 200× and 400× in oil immersion (Nikon, Japan).

#### Histomorphometric Analysis by Means of Stereological Method

A stereological analysis according to Delesse’s principle was applied to all specimens to compute volume fractions of the tissue components of the regenerated area within the trabeculae of the implant, expressed as the percentage of the total volume in the portion of the defect (Canullo et al., 2016).

Ten photos per slide were acquired at 100× magnification. On each photo, a point-counting procedure with a 100 test point grid (PhotoshopCS5Extended; Adobe, San Jose, CA) was used to obtain the proportions of the specimen occupied by every regenerated tissue (lamellar bone, woven bone, osteoid, medullary spaces) and by the scaffold. The mean values out of the ten photos were computed for each tissue.

#### Histomorphometric Analysis

The percentage ratio between the linear contact of the newly formed tissue including lamellar bone, woven bone and osteoid within the implants and the total perimeter of the implants was calculated on the same slides used for the stereological analysis in order to quantify the amount of osseointegration using a dedicated navigation software (Image J, NIH; Varoni et al., 2015). A mean value over the 10 photos was then computed and an average tissue to implant contact (TIC) was obtained for each section (Fabbro et al., 2017; Ragone et al., 2020).

Two experts analyzed in blind 20 photos of the same samples at a total magnification of 400× in order to evaluate the inflammatory population using a semi-quantitative score following ISO 10993-6:2007 that specifies methods for the assessment of the local effects of implanted biomaterials intended for use in medical devices. No statistically significant differences were found between the two datasets (paired *T*-test, *p* > 0.05). Once the method was set, semi-quantitative and qualitative analyses were performed on 10 fields of the treated area at a total magnification of 400×. On each section, a global score between 0 and 4 was given to each of the following parameters: presence/absence of inflammatory infiltrate, fibrosis, fatty infiltrate, and necrosis.

### Bone to Implant Contact (BIC) by Means of Backscattered Electron Microscopy (BSE-SEM)

Backscattered electron microscopy (BSE-SEM) offers a considerable insight into the mineralized tissues at the implant-bone interface (Shah et al., 2019). BSE-SEM is particularly useful to discern one material from another, since the yield of the collected backscattered electrons increases monotonically with the specimen’s atomic number, thus allowing to distinguish elements with atomic number differences of at least three (Rocchietta et al., 2007). Specifically, into the various gray areas, BSE-SEM can interpret bone remodeling sequences from islands of calcifications appearing as non-organized agglomerates of hydroxyapatite crystals till a more structured and organized lamellar bone. Therefore, BSE-SEM was used to compute bone to implant contact (BIC) as the percentage ratio between the linear contact of the new calcified matrix within the implants and the total perimeter of the implants. The histological sections prepared for stereological analyses were then observed under BSE-SEM without additional fixation and previous coating of gold film in order to evaluate the level of mineralization of the regenerated tissue within the trabeculae of the implant (TESCAN LYRA3 SEM, Tescan, Czechia). Three images per section were randomly acquired within the trabeculae of the implant setting the field area at 5 mm × 5 mm. The image analysis to calculate the BIC was performed as previously described for TIC.

### Statistical Analysis

Statistical analyses were performed using the GraphPad Prism 5 software (GraphPad Software, Inc., La Jolla, CA, United States). Shapiro–Wilk test assessed the normal data distribution. Data obtained by cytotoxicity tests were analyzed using one-way analysis of variance (ANOVA) coupled with Bonferroni’s *post hoc* test. Histological stereological data, BIC and local effects parameters were analyzed using two-tailed paired *t*-test. Values of *p* < 0.05 were considered statistically significant. Data are expressed as the mean ± SE. For the *in vivo* study, the sample size was calculated by means of the Mead’s resource equation for which *E* = (12 experimental units for each anatomical site – 1) – (2 treatment groups – 1) = 10.

## Results

### Chemical, Physical and Morphological Characterization of Cs Peptide-Enriched SF Sponges

Prior to the preparation of the TT+SF/Cs composite devices for the *in vivo* study, the properties of the SF sponges loaded with variable amounts of Cs peptides, ranging from 2.5% w/w to 25% w/w (with respect to SF) were investigated to assess whether the chemical and physical properties of SF were affected by the addition of the peptide fraction.

Table 1 lists the amino acid composition of SF and Cs alone, and of SF sponges containing 5, 10, and 25% w/w of Cs peptides. SF showed the typical amino acidic pattern, characterized by the presence of large amounts of simple amino acids, i.e., glycine, alanine, and serine. The Cs peptides displayed higher amounts of polar and bulky side chain amino acids, in particular aspartic acid, glutamic acid, lysine, and arginine. The addition of Cs peptides resulted in an overall increase of polar and bulky side chain amino acids of Cs-enriched SF sponges (Supplementary Figure 2A).

**TABLE 1:** Amino acid composition of silk fibroin (SF), Cs peptides, and SF sponges with 5, 10, and 25% w/w peptides with respect to SF.

AA (μmol/g)	SF	Cs	SF + 5% Cs	SF + 10% Cs	SF + 25% Cs
Asp	263	574	273	317	299
Thr	135	248	167	141	152
Ser	1,597	720	1,613	1,558	1,423
Glu	142	320	212	191	194
Pro	73	253	69	81	90
Gly	6,705	4,031	6,459	6,554	6,084
Ala	4,430	2,492	4,342	4,357	3,961
Cys	0	0	0	0	0
Val	323	515	337	361	361
Met	0	0	0	0	0
Ile	142	205	114	155	126
Leu	103	235	95	116	118
Tyr	717	950	740	734	753
Phe	59	88	78	82	84
Lys	56	126	58	74	79
His	27	73	28	21	25
Arg	68	131	61	81	56

**FIGURE 2:**
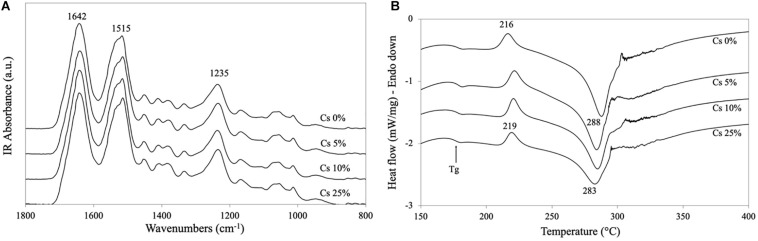
Physical properties of silk fibroin (SF)/Cs sponges. **(A)** attenuated total reflection (ATR)-fourier transform infrared (FTIR) spectra, and **(B)** differential scanning calorimetry (DSC) thermograms of SF sponges without Cs peptides and with different amounts of peptides (5, 10, and 25% w/w).

The ATR-FTIR spectra evidenced the amorphous character of the sponges as indicated by the position and shape of Amide I (broad peak at 1,642 cm^–1^), Amide II (peak at 1,515 cm^–1^, with a strong shoulder at higher wave numbers), and Amide III (peak at 1,235 cm^–1^) bands (Figure 2A). The addition of Cs peptides up to 25% w/w did not change the FTIR spectra, meaning that the SF macromolecules in all freeze-dried sponges kept their amorphous three-dimensional arrangement. The DSC (thermogram) of the SF sponge without peptides showed the typical profile of an amorphous SF material (Figure 2B) with a glass transition temperature (Tg) at about 178°C, an exothermic peak at 216°C (SF crystallization), and a strong endothermic transition at 288°C melting/degradation of SF. As the content of Cs peptides increased, the thermal profiles essentially remained unchanged, but the onset of Tg slightly shifted to a lower temperature (Supplementary Figure 2B), the crystallization peak moved to a higher temperature, the melting degradation peak shifted to a lower temperature, and the enthalpy associated with the crystallization and melting/degradation transitions decreased (Supplementary Figure 2C,D, respectively). These thermal details are attributable to the strengthening of the amorphous character of the material, as well as to the dilution of the high molecular weight SF component caused by the addition of the low molecular weight Cs peptides.

**FIGURE 3:**
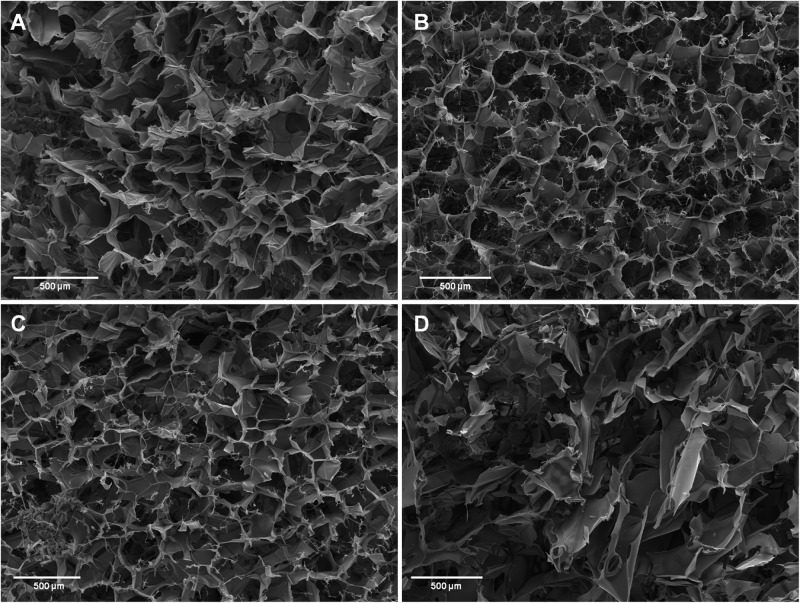
Cross-section morphology of silk fibroin (SF) sponges by scanning electron microscopy (SEM) analysis. **(A)** SF sponge without peptides. Magnification 120× (scale bar 500 μm) **(B–D)**. SF sponges enriched with 5, 10, and 25% w/w Cs peptides, respectively. Magnification 100× (scale bar 500 μm).

The cross-sectional morphology of SF sponges without and with 5, 10, and 25% w/w of Cs peptides is shown in Figure 3. Up to 10% w/w of Cs peptides, the sponges did not show significant morphological changes of the polygonal-shaped voids. The pored area, estimated by SEM measurements (Supplementary Figure 3), slightly increased with respect to that of the neat SF sponge.

As the amount of Cs peptides increased up to 25% w/w, the porous structure became irregular and the pored area increased further. It is worth noting that handling this sample was quite difficult due to its high fragility, as if the high amount of peptides had prevented the SF chains from compacting during the cold coagulation phase, before freeze-drying.

### *In vitro* Biocompatibility of SF Sponges

The poor handling properties of SF sponges with the highest amount of Cs peptides (i.e., 25% w/w) precluded their use as cell substrate for the *in vitro* biocompatibility study. Therefore, analogously to what previously published on Cs peptides loaded on dense collagen gel (Marelli et al., 2014) or on electrospun SF matrices (Griffanti et al., 2018) as polymeric carriers, 10% w/w was selected as the maximum amount of Cs peptides to be loaded on SF sponges.

**FIGURE 4:**
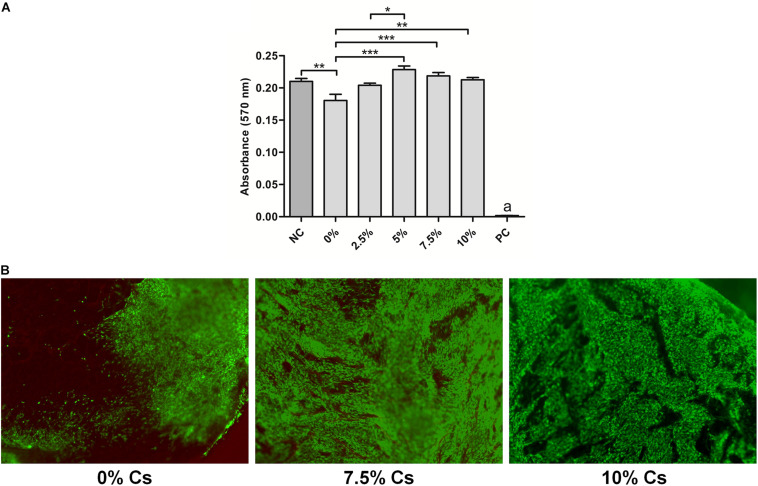
Viability assays. **(A)** MTT assay on NIH-3T3 fibroblasts indirectly cultured with silk fibroin (SF) sponges without peptides (0%), with different concentrations of peptides (Cs 2.5, 5, 7.5, and 10% w/w) or cultured in fresh medium as a negative control (NC). The positive control (PC) represents cells treated with Triton-X100. Significance: *p < 0.05; **p < 0.01; a, ***p < 0.001. **(B)** Live&Dead assay of NIH-3T3 fibroblasts directly cultured onto SF sponges without peptides (0%), with different concentrations of peptides (Cs 7.5 and 10%).

Assessing the biocompatibility of SF sponges, a significantly higher viability was measured in NIH-3T3 fibroblasts cultured in transwell with SF sponges with 5, 7.5, and 10% w/w Cs peptides respect to cells cultured with SF sponges without Cs peptides (Figure 4A).

Based on these results, the cell viability test was performed by culturing cells directly only on SF without peptides and on those enriched with 7.5% and 10% w/w peptides. The Live&Dead assay confirmed that the tested biomaterial had a good biocompatibility with a scarce presence of dead cells. A complete cell colonization of the scaffold was observed in 7.5% and 10% w/w Cs peptide-enriched SF sponges. Conversely, SF sponges without Cs peptide enrichment were characterized only by a peripheral cell distribution (Figure 4B).

### Morphological Characterization of TT+SF/Cs Composite Devices

**FIGURE 5:**
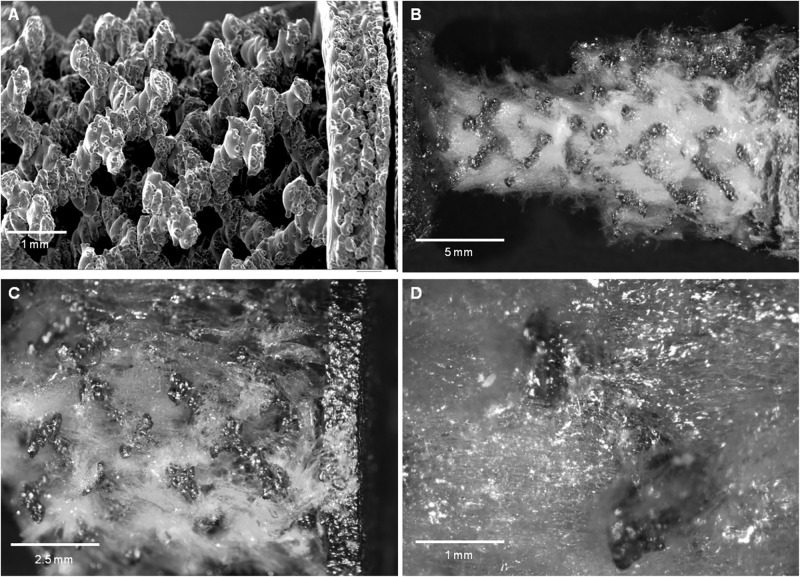
Trabecular titanium (TT) and TT+silk fibroin (SF)/Cs implants. **(A)** scanning electron microscopy (SEM) image of the trabecular structure of the TT device before impregnation with the SF/Cs sponge. Magnification 50× (scale bar 1 mm). **(B–D)** Optical Microscopy images at increasing magnification 4×, 10×, 40×, respectively (scale bars: 5, 2.5, and 1 mm, respectively) of the TT+SF/Cs implant device where the surface spongy material was removed. The trabecular section of the device is fully infiltrated by the spongy material. No macroscopic voids are visible.

Based on the results of the *in vitro* biocompatibility assay, the TT device (Figure 5A) was filled with the SF sponge loaded with 10% w/w Cs peptides. The TT device was placed in a mold containing the 10% w/w SF/Cs solution, and freeze-dried to generate the TT+SF/Cs device with the macroporous trabecular structure completely immersed in the polymer sponge. The thin polymer skin formed at the interface between the spongy material and the wall of the mold was gently removed, leaving a device with the macroscopic appearance shown in Figure 5B.

To verify whether the SF/Cs sponges filled the voids of the TT structure, the superficial spongy coating was removed until the trabecular material was exposed. As shown in Figures 5B–D, the voids were completely filled by a homogeneous spongy phase. No gaps were detected between the sponge and the metal structure. The roughness of the metal surface (Figure 5A) increased the contact area and resulted in a good adhesion of SF to the titanium structure.

### *In vivo* Study and Validation of TT+SF/Cs Composite Device

**FIGURE 6:**
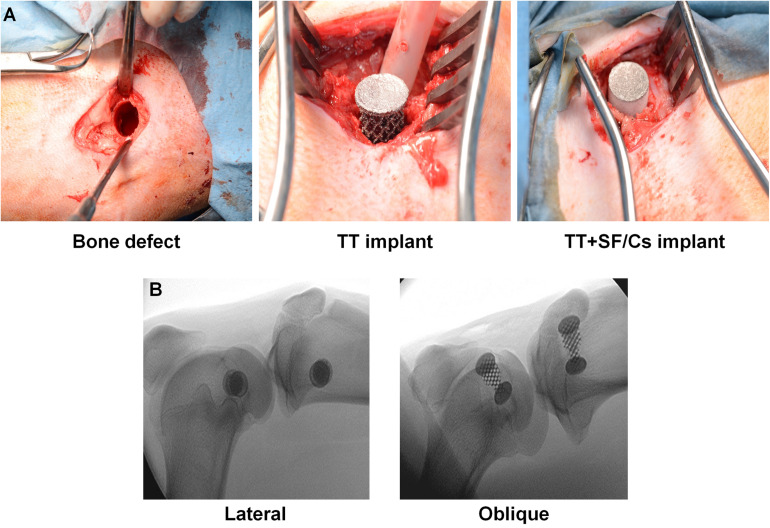
Surgical procedure and fluoroscopic imaging. **(A)** Creation of the bone defect (11 mm Ø × 20 mm h); trabecular titanium (TT) and TT+ silk fibroin (SF)/Cs implants fixation within the defects by press fit. **(B)** Representative lateral and oblique fluoroscopic projections of implanted tibia and femur.

Six sheep were randomly implanted with TT or TT+SF/Cs in bone defects in the femur and tibia to avoid a location bias (Figures 6A,B)

Two hours after surgery, all animals were fully weight-bearing. Neither postoperative complications nor signs of infections were observed during the follow-up period, and sheep conducted a normal daily activity during the entire 8 weeks of follow-up, until animal sacrifice.

### Qualitative and Quantitative Histological Analyses

**FIGURE 7:**
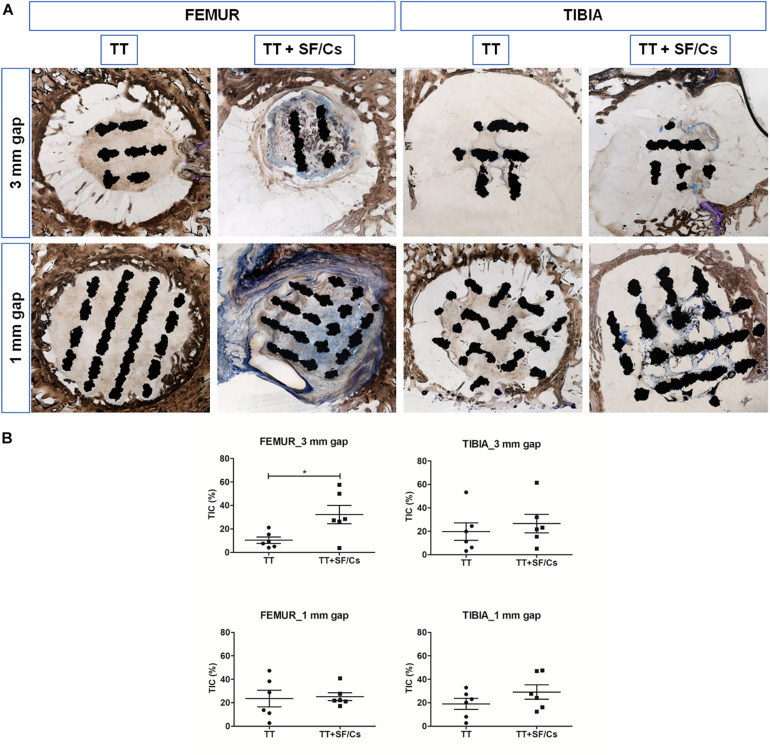
Histological and histomorphometric analyses of trabecular titanium (TT) and TT+ silk fibroin (SF)/Cs implants in femoral and tibial sites. **(A)** Overview of one representative section per group. Two months after surgery, new bone matrix in variable stages of mineralization (stained in blue) have surrounded and enclosed the TT+SF/Cs implant, mostly in femoral sites within both gaps. Toluidine Blue and Pyronin Yellow staining. **(B)** Histograms reporting the tissue implant contact (TIC) as the percentage of newly formed tissue in contact with the implant surface. *p < 0.05. Black colored circles refer to TT samples, Black colored square shapes refer to TT+SF/Cs.

Qualitative and quantitative histological analyses were performed to assess the deposition and maturation of newly formed bone tissue within the titanium implant trabeculae and at the bone-implant interface. From a qualitative point of view, the TT+SF/Cs implants seemed to be characterized by more new bone matrix compared to the sites implanted with TT alone both in the case of small (1 mm) and large (3 mm) gaps, and particularly in the femur (Figure 7A). The quantitative evaluation of the newly formed tissue in contact with the implant (TIC) confirmed the higher trend of TT+SF/Cs vs. TT alone in filling the spaces among the trabeculae of the implant. This effect was more evident in the femur in the case of large gaps (*p* < 0.05; Figure 7B).

**FIGURE 8:**
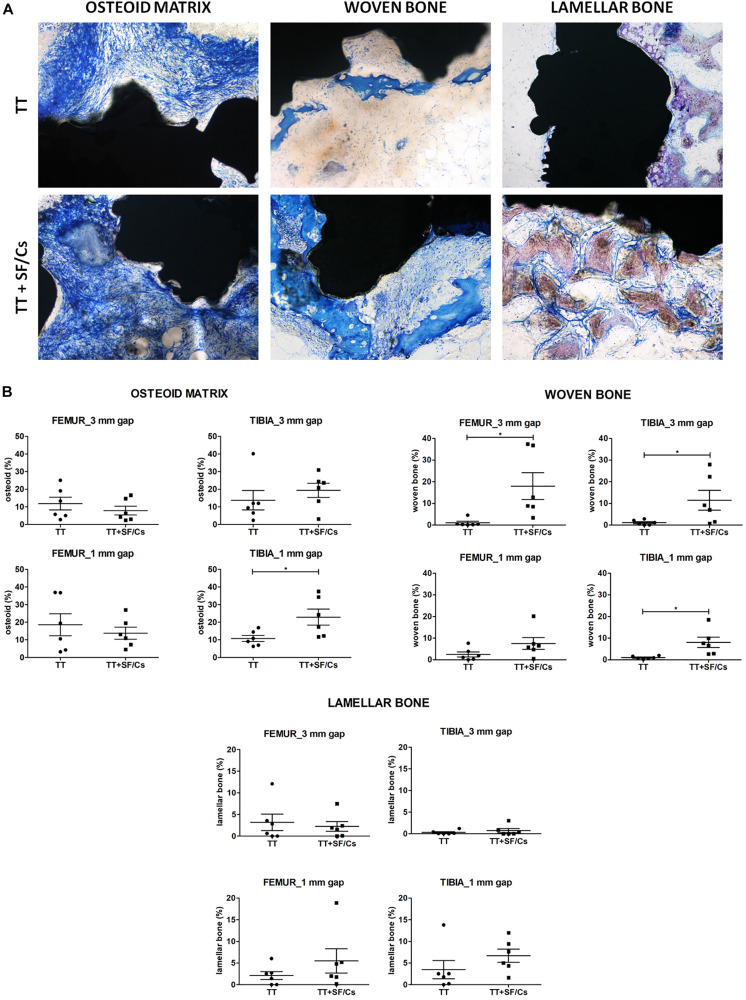
Qualitative and quantitative histology. **(A)** Representative histological sections reporting the stages of the newly bone matrix organization: the low mineralized tissue (osteoid and woven bone) is stained in blue, the more mineralized tissue (mature bone) is stained in purple/brown. Osteoid matrix presents as a three-dimensional network of collagen bundles, while the woven bone is characterized by a compact structure of collagen fibers resembling primitive trabeculae not yet completely mineralized. The lamellar bone is formed by islands of mineralized interconnected by bridges of not mineralized bone matrix. Toluidine Blue and Pyronin Yellow staining. Total magnification 100×. **(B)** Histograms report the semi-quantitative histomorphometric analysis of the three different stage of bone matrix organization: osteoid matrix, woven bone, lamellar bone. *p < 0.05. Black colored circles refer to TT samples, Black colored square shapes refer to TT+SF/Cs.

In the qualitative assessment of newly formed tissue, we focused on the presence of bone tissue at different maturation stages. In general, osteoid matrix was characterized by dense bundles of collagen fibers forming a three-dimensional network. A more organized tissue, the woven bone, was present as islands of compact connective matrix in the phase of mineralization. Remarkably, the qualitative analysis of the samples already highlighted a more abundant woven bone development in the defects containing the TT+SF/Cs implants. Finally, the last stage of the tissue maturation appeared as areas of mature bone layered to form the lamellar bone (Figure 8A). The histomorphometric analysis showed lower osteoid matrix deposition in TT+SF/Cs compared to TT in femurs, while an opposite trend was found in the tibial defects, in which greater osteoid matrix formation was detected in TT+SF/Cs compared to TT, especially in the small gaps (*p* < 0.05). The histomorphometry also confirmed higher woven bone formation within the sites implanted with TT+SF/Cs compared to TT at the level of both femur and tibia (*p* < 0.05). Despite no significant differences in the amount of new lamellar bone were found between TT and TT+SF/Cs implants, in the small gaps of both femoral and tibial sites, the mature bone seemed to have an increasing trend in TT+SF/Cs composites than in TT alone (Figure 8B).

### BSE-SEM Analyses

**FIGURE 9:**
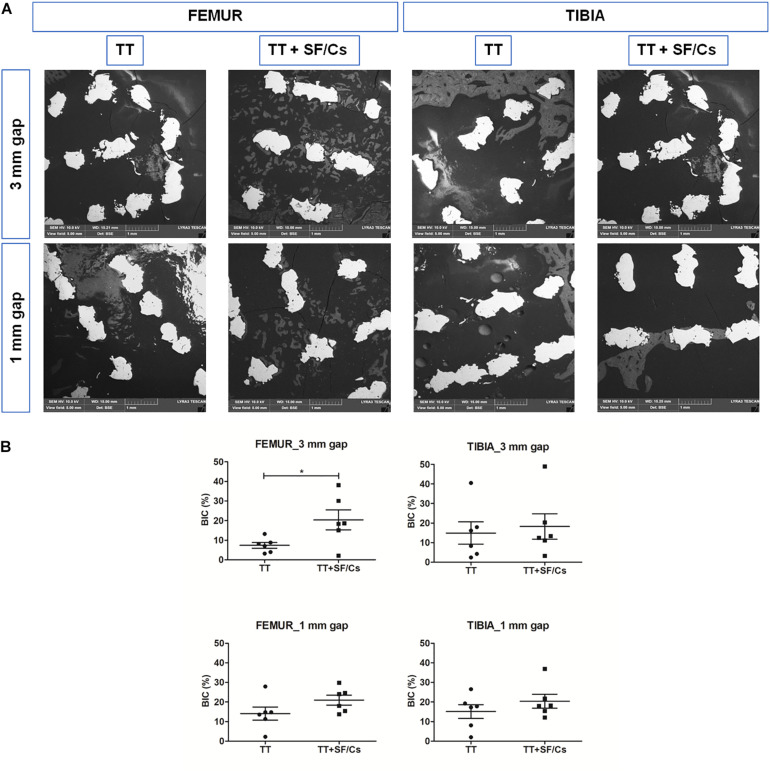
Backscattered electron microscopy (BSE)-scanning electron microscopy (SEM) analyses of trabecular titanium (TT) and TT+ silk fibroin (SF)/Cs implants in femoral and tibial sites. **(A)** Overview of one representative section per group. Two months after surgery, calcified bone/hydroxyapatite crystals in gray are present in the area that surrounded the TT+SF/Cs implant trabeculae (in white), mostly in femoral sites within both gaps. **(B)** Histograms reporting the bone implant contact (BIC) as the percentage of newly calcified bone in contact with the implant surface. *p < 0.05. Black colored circles refer to TT samples, Black colored square shapes refer to TT+SF/Cs.

The backscattered SEM analysis showed various tissue densities on a gray scale and SEM was used to further corroborate the results provided by the histomorphometric analysis. The titanium implant, consisting in a very dense metal structure, was seen as white areas. The mineralized matrix (lamellar bone) appeared as gray structures, while any soft tissue (bone marrow) or poorly mineralized matrix (osteoid matrix, woven bone) resulted as black areas. BSE-SEM images revealed the presence of several nuclei of calcified tissue within the trabeculae of the implant, in the femur sites treated with TT+SF/Cs especially in the 3 mm gap defects. In the tibia sites treated with TT+SF/Cs, some areas of organized and calcified tissue were visible among the peripheral trabeculae of the implant. In the TT sites of both tibia and femur, calcified tissue presented as patches of dense agglomerates of hydroxyapatite crystals (Figure 9A). The newly calcified bone matrix in contact with the implant trabeculae (BIC) was significantly higher in the TT+SF/Cs vs. TT alone in the femoral large gaps (*p* < 0.05; Figure 9B). Moreover, BIC values reflected the same trends of the TIC, although lower values were found due to the selective detection of highly calcified matrix by BSE-SEM.

### Biological Evaluation of TT+SF/Cs Composite: Local Effects After Implantation

**FIGURE 10:**
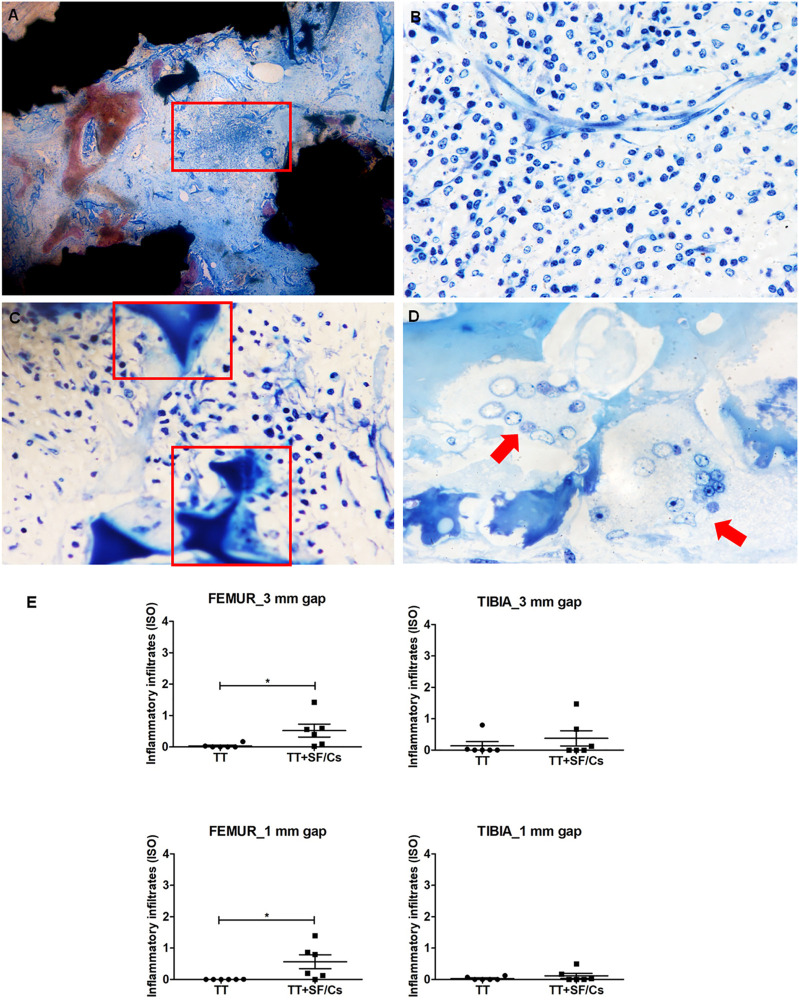
Inflammatory reaction in trabecular titanium (TT)+silk fibroin (SF)/Cs sites, and histograms. **(A)** Localization of the inflammatory infiltrate within the core of the samples (red box). **(B)** Subacute reaction with various inflammatory cells (granulocytes, plasma cells, and lymphocytes) around blood vessels. **(C)** Osteoblast- and osteoclast-like cells lining along new bone trabeculae (red boxes). **(D)** Multinucleated giant cells (red arrow) in the proximity of the remodeling site. Toluidine Blue and Pyronin Yellow, magnification 40× **(A)**, 400× **(B,C)**, 600× **(D)**. **(E)** Semi-quantitative analysis of the inflammatory infiltrates, *p < 0.05. Black colored circles refer to TT samples, Black colored square shapes refer to TT+SF/Cs.

The local effects of implants were assessed based on the ISO 10993-6:2007 tables. This assessment detected no areas of necrosis or fibrosis in any samples. In some samples of both TT and TT+SF/Cs implants, a tissue reaction was observed in the core of regenerated sites (Figure 10A, red box), consisting in subacute infiltrate of inflammatory cells (granulocytes, plasma cells, and lymphocytes; Figure 10B) along with osteoblast- and osteoclast-like cells lining onto the newly formed bone trabeculae (Figure 10C, red boxes). Furthermore, scattered multinucleated giant cells (red arrow) were observed in some of the TT+SF/Cs implants as a response of resorption to the SF -like remnants (Figure 10D). Despite the semi-quantitative analysis found a greater inflammatory response in the TT+SF/Cs treated defects compared to the TT in femurs (Figure 10E), for all the analyzed groups, the values were below the grade 1 of the ISO 10993-6:2007 scale indicating a very low inflammatory response, as reported in the histograms (Figure 10). No statistically significant differences in the inflammatory responses between TT+SF/Cs and TT implants were observed in the tibia sites.

## Discussion

The enhancement of bone-to-implant anchorage following uncemented arthroplasty is a desirable outcome in prosthetic surgery. The aim of this study was to investigate the enhancement of the osseointegration of macroporous titanium implants loaded with SF/Cs sponges (TT+SF/Cs) compared to pure titanium implants (TT) in small and large *in vivo* gap models, taking advantage of the peculiar properties of SF/Cs in supporting the nucleation of carbonated hydroxyapatite and inducing osteogenic differentiation of bone marrow progenitor cells (Marelli et al., 2012, 2014). Here, for the first time, we combined highly porous TT with a SF/Cs sponge to functionalize the titanium implants. The hypothesis of this study is that the hybridization of SF/Cs sponges with TT can generate mechanically strong, biocompatible, and bioactive devices able to solve open questions in terms of lack of mineralization and limited osseointegration of titanium prostheses in the case of bone-to-implant interfacial gaps. In fact, combining many functional features in one single device is mandatory to achieve positive outcomes during the process of bone-implant integration in the short-to-medium term and guarantee the long-term fixation of implants.

The surface modification of implants with SF has been recently reported, demonstrating that the use of SF facilitated the initial cell adhesion, improved cell spreading and induced better mineralization without eliciting a significant immune response. However, to ensure the stability of the fibroin coating, the pretreatment of titanium surface by silanization with 3-aminopropyltriethoxysilane (APTES) was necessary (Naskar et al., 2014). Remarkably, the herein proposed TT+SF/Cs devices were manufactured by using an entire aqueous process, without employing chemicals that might leave potentially harmful residues. The incorporation of Cs peptides into a SF aqueous solution, followed by freeze-drying, produced this novel biomaterial preserving the physico-chemical, mechanical, and biological properties of pure SF. The digestion of SF with α-chymotrypsin has been reported to generate a set of fibroin-derived polypeptides with a well-defined structure and charge (Marelli et al., 2012). Thanks to the cleavage specificity of α-chymotrypsin, Cs peptides, i.e., the peptides purified from the chymotryptic supernatant, are enriched in amino acid sequences deriving from the so-called “amorphous domains” of the SF chain that regularly alternate with the highly repetitive “crystalline domains” rich in glycine, alanine, and serine, in line with the literature (Zhou et al., 2000, 2001). Notably, the high content of carboxylated amino acids (Asp, Glu) confers a strong anionic character to these peptides (Marelli et al., 2012). The amino acid analysis showed that the addition of Cs peptides to SF sponges increased the relative amount of acidic amino acids, thus imparting a stronger electronegative character to the resulting SF/Cs mixed material, as also stated elsewhere (Griffanti et al., 2018).

In line with previously reported characterization of SF, the FTIR and DSC data obtained on neat SF and SF/Cs sponges returned the image of an essentially amorphous SF material, with prevailing random coil molecular conformation (Magoshi et al., 1979; Tsukada et al., 1994b; Taddei and Monti, 2005; Boulet-Audet et al., 2015). In this study, any attempt aimed at consolidating SF sponges to make them water insoluble, for example by immersion in a suitable solvent like methanol, which is known to render SF materials insoluble in water by inducing β-sheet crystallization of SF chains (Tsukada et al., 1994b), was not taken into account because the primary goal was to make the osteogenic Cs peptides readily and quickly available at the site of implantation to start the osteogenic processes as soon as possible. Indeed, we verified the kinetics of the dissolution of non-consolidated SF sponges when immersed in water for 1 and 24 h (data not shown). This evaluation showed that sponges containing 10% w/w Cs peptides had a loss of about 50% of the original weight after 1h of immersion, afterward, the dissolution rate sharply decreased with an additional 5% of mass loss after 24 h. This suggests that the dissolution is rapid at the very beginning (1 h) of fluid contact, becoming stable over time (24 h), as also supported by the persistence of fibroin sponges *in vivo* 2 months after implantation. This choice was underpinned by the fact that an early 2-month time point was selected for the *in vivo* test to better identify the initial bone response to the implanted materials. However, it cannot be excluded that longer-term *in vivo* studies might require the development of devices capable of releasing the active Cs peptides more slowly and more continuously over time. In this case, a treatment leading to SF sponge consolidation may suitably slow down the rate of release of Cs peptides owing to the slower rate of degradation of the Cs-carrier SF material (Guo et al., 2020; Li et al., 2020). The cross-sectional morphology of SF sponges without and with increasing amounts of Cs peptides up to 10% w/w was characterized by the presence of polygonal voids, separated by thin SF walls. This morphological features agree with previously reported data (Tsukada et al., 1994a). The addition of larger amounts of Cs peptides (i.e., 25% w/w) not only disturbed the morphological regularity of the sponges, but also made them almost unsuitable to handling due to excessive material fragility.

To generate the TT+SF/Cs composite, a macroporous TT (pore size: 1,200 μm) was selected to favor the compenetration between the two materials. Additionally, the use of titanium implants with wide porosity has been shown to enhance both the biological anchorage to the bone and the bone implant contact area without decreasing the mechanical properties (Regis et al., 2015). Our results showed that the bioactive SF material was not confined to the titanium surface, but filled the voids of the TT, thus providing a continuous biocompatible substrate for cells to populate the implant and to initiate the remodeling process that leads to bone regeneration. Indeed, the main hypothesis underlying this study was that the osteoinductive features of Cs peptides could favor the outward bone growth from the TT+SF/Cs implants, which commonly lacks in unloaded titanium implants, thus optimizing the initial fixation of the prosthetic device.

To test the efficacy of TT+SF/Cs devices in enhancing the early bone ingrowth, sheep were chosen as a relevant large animal model of multiple bone defects due to bone structure and osseointegration resemblance with humans (Aerssens et al., 1998; Martini et al., 2001; Gardel et al., 2014; Lovati et al., 2016). Small (1 mm) and large (3 mm) gaps at the bone-implant interface were studied to resemble different clinical scenario encountered in the case of revision surgery. The advantages of this study lay in the direct comparison of pure TT, as control implants, and TT+SF/Cs implants within each animal, thus reducing the internal biological variability and the number of animals used. Despite a single time point could represent a limitation of the study, based on our previous experience (Lovati et al., 2016), we selected an early 2-month time point to better identify the initial bone response to the treatment, thus avoiding the masking effects of bone deposition that typically occur in this species at later time points.

Qualitative and quantitative histological analyses were performed to assess the deposition of newly formed tissue within the titanium implant trabeculae and to highlight the different phases of maturation.

The BSE-SEM analysis was performed to specifically discriminate the mineralized matrix in contact with the implant. This technique is known to specifically detect highly calcified matrix, but it is unable to identify the osteoid due to its low level of calcification (Shah et al., 2019). By contrast, the specific histological dichromic staining used to compute the tissue fractions by optical microscopy enabled to sharply distinguish osteoid matrix and highly mineralized bone (Fabbro et al., 2017; Varoni et al., 2018; Shah et al., 2019; Ragone et al., 2020).

In defects evaluated after 2 months of healing, the new bone matrix was composed predominantly by osteoid and woven bone, and a limited amount of lamellar bone, as expected due to the early time point. BIC showed a statistically significant augmentation in femur large gaps treated with TT+SF/Cs than in TT implants. The BIC analysis was performed to visualize the amount of new calcified bone matrix deposited in contact with the implant trabeculae in order to focus on the properties of the implant surface. According to our previous study (Lovati et al., 2016), BIC and new bone matrix deposition within the implant trabeculae were significantly higher in the femoral site rather than tibia, particularly in 3 mm gap sites. The characterization of the various components of the new bone matrix by means of the current dichromic staining highlighted a difference in the healing pattern between TT+SF/Cs and TT implants, showing a more advanced phase of mineralization in the defects implanted with TT+SF/Cs. Indeed, the composition of the new bone matrix in the TT sites was mainly made by osteoid and a limited amount of mature bone, while in the TT+SF/Cs an additional significant increase of woven bone was detected in both femoral and tibial 3 mm gap sites and in tibial 1 mm gap sites. Also, a trend toward an increasing amount of new lamellar bone was found in small gap defects of both femur and tibia implanted with TT+SF/Cs. Hence, histological analyses suggest that the treatment tested in the present study seems to favor the mineralization process in the early stages of healing.

In general, the present study belongs with the series of diverse approaches aimed at improving implant osseointegration by applying for instance porous titanium implants coated with hydroxyapatite nanoparticles in poly-DL-lactic acid or titanium loaded with BMP-2 and testing the implant performances in a sheep model (Jensen et al., 2010; Hunziker et al., 2016). Both studies demonstrated that gaps lacked either the bony bridging or the new bone formation within the empty porous spaces of titanium implants during the early phase of osseointegration. The absence of effects on early fixation was also reported in ovine femoral gaps implanted with titanium alloy enriched with hydroxyapatite/collagen, bone marrow aspirate and demineralized bone matrix or electromechanically deposited CaP (Chen et al., 2011; Babiker et al., 2012; Babiker et al., 2016). Good results were only obtained in the late-term fixation of titanium implants coated with CaP combined with mesenchymal stromal cells or treated with anodic spark deposition in similar models (Bertollo et al., 2015; Garcia-Gareta et al., 2015). In this scenario, the proposed TT+SF/Cs composite favored the osseointegration of implants in a very early stage of the fixation process, thus representing a significant step forward in the field of prosthetic surgery with many potential clinical applications. Based on the encouraging results obtained in terms of implant osseointegration and new bone deposition, in view of the clinical application of TT+SF/Cs composite, the local effects of implants were also assessed, based on the ISO 10993-6:2007 tables. Based on the histomorphometric results, the inflammatory reaction to TT+SF/Cs did not seem to interfere with tissue maturation. The mild presence of inflammatory infiltrates in TT+SF/Cs series was expected at the investigated time point compared to inactive pure TT implants because of the progressive resorption of SF. As future steps, it would be interesting to investigate whether the SF treatment of titanium surfaces could also prevent the release of corroded particles in the long term, that are known to produce inflammation, fibrosis and necrotic tissue leading to the aseptic loosening of the prosthesis (Petersen, 2014).

Overall, the present results demonstrated that the combination of a SF sponge enriched with anionic fibroin-derived Cs peptides and porous titanium implants induced an outward bone matrix growth that could be a great advantage to enhance the osseointegration of prosthetic implants. Moreover, thanks to the osteoconductive properties of SF/Cs sponges, the inward bone deposition of native bone toward the implants was also achieved. More importantly, the approach proposed in this study by combining SF/Cs sponges within titanium implants could be extended to different types of porous metals and other materials commonly used to generate orthopedic prostheses in order to improve the early bone fixation and to prevent the aseptic loosening in the field of prosthetic surgery.

## Data Availability Statement

All datasets generated in this study are included in the article/Supplementary Material.

## Ethics Statement

The animal study was reviewed and approved by Lazzaro Spallanzani Institute Animal Care and Use Committee (IACUC; Permit No. 454/2015-PR).

## Author Contributions

AL, SL, AA, GF, FS, and MM contributed to conception and design of the study. AL, SL, MBo, and GT performed the *in vitro* and *in vivo* study. AA, MBi, and GF synthesized and characterized silk fibroin materials. EC and CD performed the histological analyses. AL wrote the first draft of the manuscript. SL, EC, CD, MBi, and GF wrote sections of the manuscript. All authors contributed to manuscript revision, read, and approved the submitted version.

## Conflict of Interest

FS is employed by the company Limacorporate S.p.A. GF is a stock owner and consultant of Silk Biomaterials Srl. AA is a stock owner and employee of Silk Biomaterials Srl. MBi is an employee of Silk Biomaterials Srl. AL, SL, AA, MBi, GF, and MM are the inventors of the Italian patent application no. 102020000004081. The remaining authors declare that the research was conducted in the absence of any commercial or financial relationships that could be construed as a potential conflict of interest.
