# Supplementary figures and images for: Peptide-Enriched Silk Fibroin Sponge and Trabecular Titanium Composites to Enhance Bone Ingrowth of Prosthetic Implants in an Ovine Model of Bone Gaps
Source: Front Bioeng Biotechnol. 2020 Oct 19;8:563203. doi: 10.3389/fbioe.2020.563203 (PMC7604365; doi:10.3389/fbioe.2020.563203)

Supplementary Material


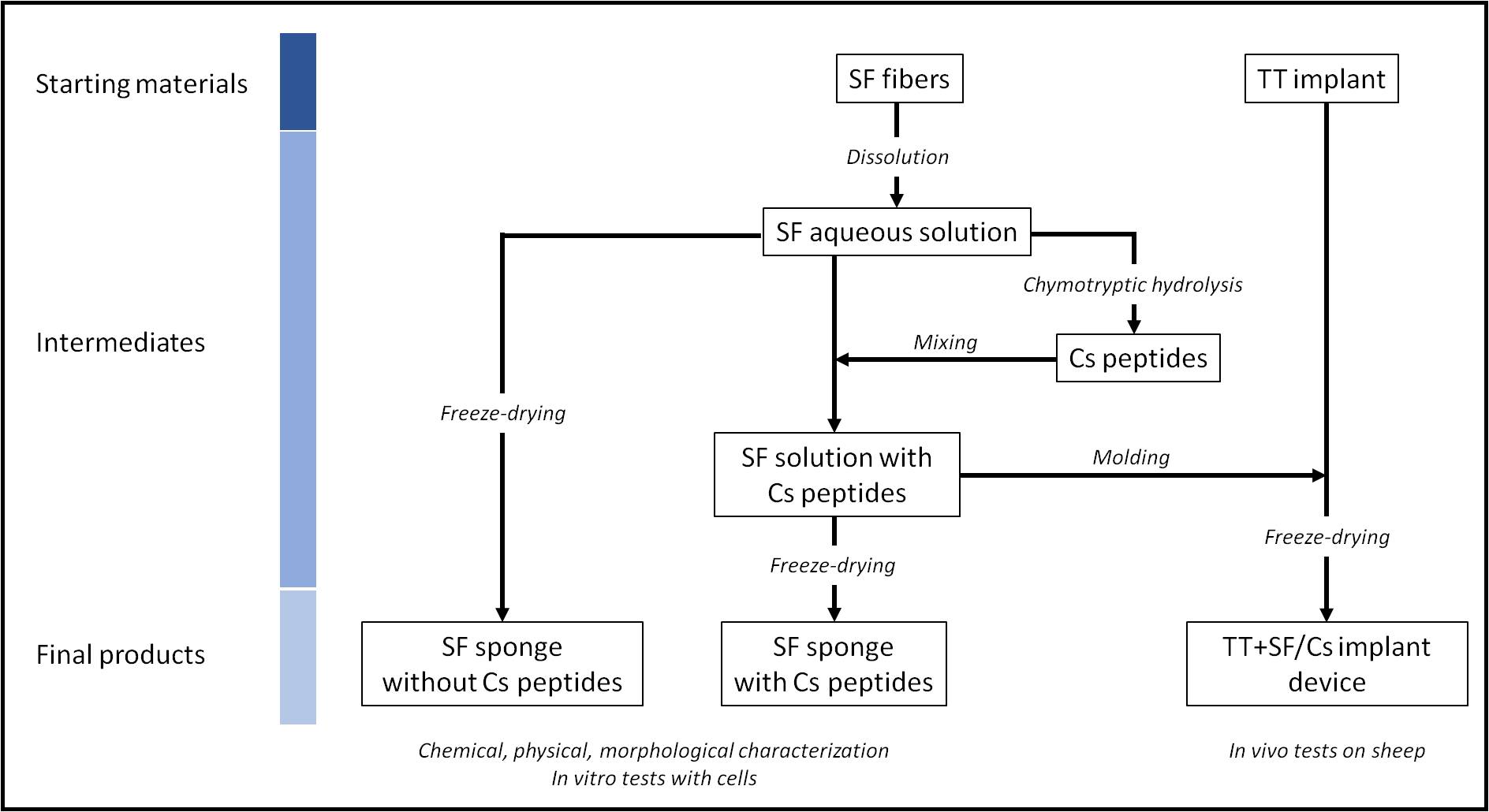


**Supplementary Figure 1.** Scheme of the materials and processes.

Supplement: Supplementary file 1 [file Table_1.DOCX]
