# Supplementary material for: Peptide-Enriched Silk Fibroin Sponge and Trabecular Titanium Composites to Enhance Bone Ingrowth of Prosthetic Implants in an Ovine Model of Bone Gaps
Source: Front Bioeng Biotechnol. 2020 Oct 19;8:563203. doi: 10.3389/fbioe.2020.563203 (PMC7604365; doi:10.3389/fbioe.2020.563203)
Supplement: Supplementary file 2 [file Table_2.DOCX]

Supplementary Material


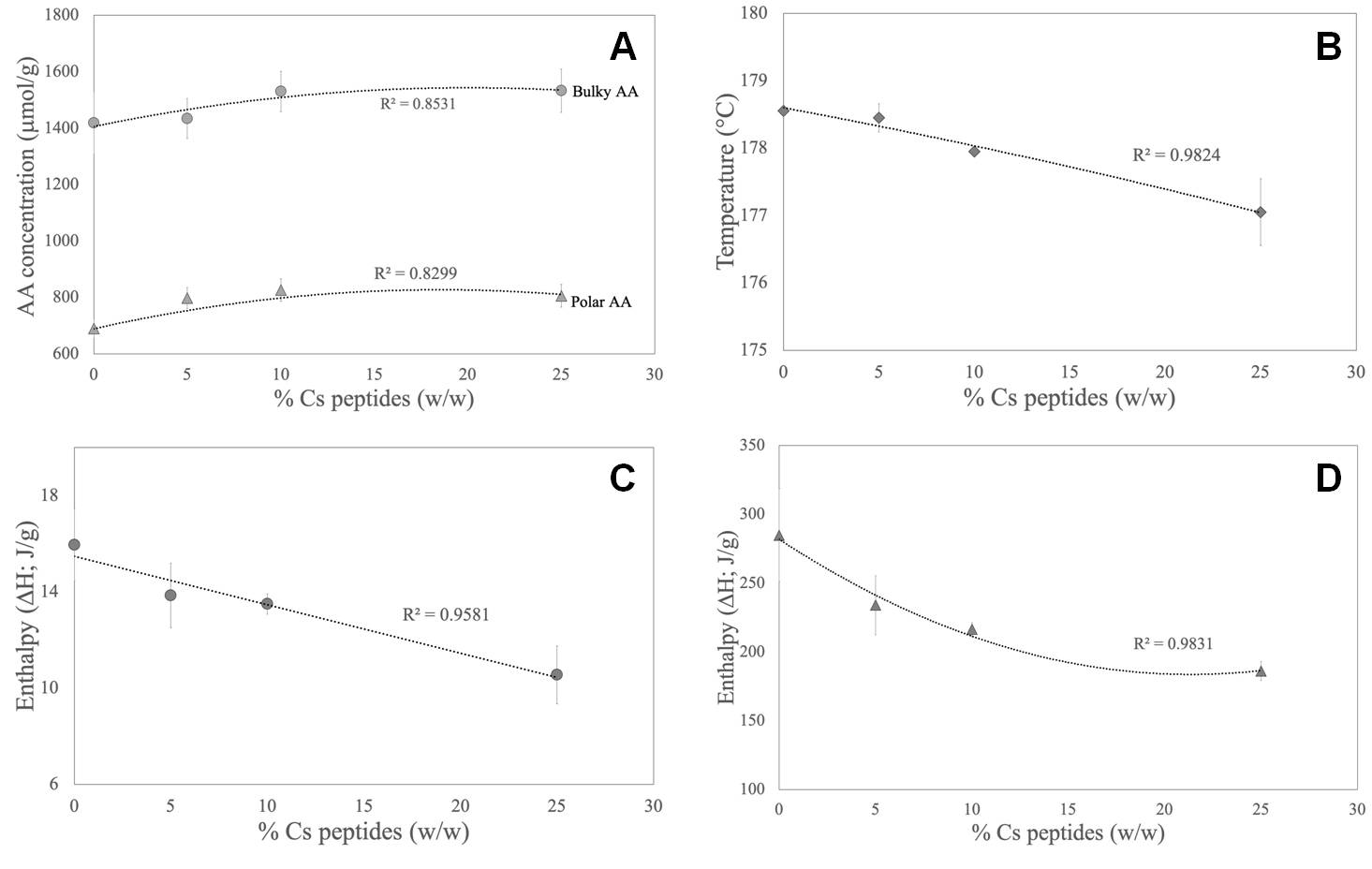


**Supplementary Figure 2.** [Chemical and physical effects of adding Cs peptides to SF sponges. (**A**) Increasing trend of bulky and polar side chain amino acids. Bulky AA: Proline, Valine, Isoleucine, Leucine (aliphatic); Tyrosine, Phenylalanine (aromatic). Polar AA: Aspartic Acid, Glutamic Acid (acidic); Lysine, Histidine, Arginine (basic). (**B**) Decreasing trend of the glass transition temperature (Tg) of SF sponges as a function of the addition of Cs peptides. (**C-D**) Decreasing trend of the enthalpy of crystallization and of the enthalpy of melting/degradation of SF as a function of the addition of increasing amounts of Cs peptides. B-D graphs were obtained from the DSC data.](FREDDI%202%20con%20figure\Lovati_Manuscript_R1_AL_GF%20bis.doc)
