# Supplementary material for: Peptide-Enriched Silk Fibroin Sponge and Trabecular Titanium Composites to Enhance Bone Ingrowth of Prosthetic Implants in an Ovine Model of Bone Gaps
Source: Front Bioeng Biotechnol. 2020 Oct 19;8:563203. doi: 10.3389/fbioe.2020.563203 (PMC7604365; doi:10.3389/fbioe.2020.563203)
Supplement: Supplementary file 3 [file Table_3.DOCX]

Supplementary Material


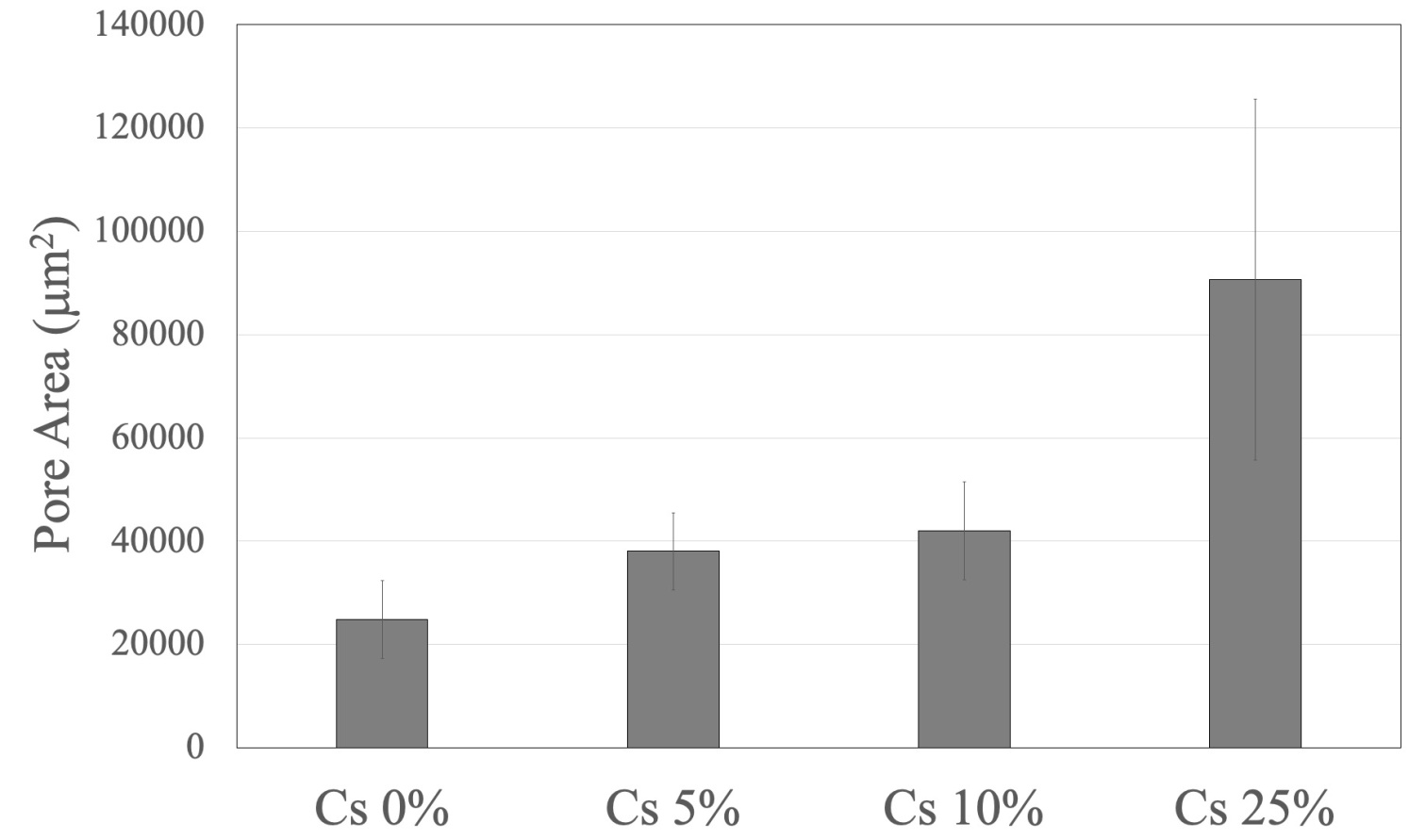


**Supplementary Figure 3.** Area of the pores of SF sponges as a function of the addition of Cs peptides (n = 50). The area was measured from the SEM images of the sponges by using the ImageJ image analysis software.
